# Genomic and Non-Genomic Regulatory Mechanisms of the Cardiac Sodium Channel in Cardiac Arrhythmias

**DOI:** 10.3390/ijms23031381

**Published:** 2022-01-26

**Authors:** Houria Daimi, Estefanía Lozano-Velasco, Amelia Aranega, Diego Franco

**Affiliations:** 1 Biochemistry and Molecular Biology Laboratory, Faculty of Pharmacy, University of Monastir, Monastir 5000, Tunisia; 2 Department of Experimental Biology, University of Jaen, 23071 Jaen, Spain; evelasco@ujaen.es (E.L.-V.); aaranega@ujaen.es (A.A.); dfranco@ujaen.es (D.F.); 3 Medina Foundation, Technology Park of Health Sciences, Av. del Conocimiento, 34, 18016 Granada, Spain

**Keywords:** cardiac sodium channel, gene regulation, cardiac arrhythmias

## Abstract

Na_v_1.5 is the predominant cardiac sodium channel subtype, encoded by the *SCN5A* gene, which is involved in the initiation and conduction of action potentials throughout the heart. Along its biosynthesis process, Na_v_1.5 undergoes strict genomic and non-genomic regulatory and quality control steps that allow only newly synthesized channels to reach their final membrane destination and carry out their electrophysiological role. These regulatory pathways are ensured by distinct interacting proteins that accompany the nascent Na_v_1.5 protein along with different subcellular organelles. Defects on a large number of these pathways have a tremendous impact on Na_v_1.5 functionality and are thus intimately linked to cardiac arrhythmias. In the present review, we provide current state-of-the-art information on the molecular events that regulate *SCN5A*/Na_v_1.5 and the cardiac channelopathies associated with defects in these pathways.

## 1. Introduction

The upstroke phase of the cardiac action potential (AP) is mainly coordinated by cardiac sodium channels, which are immediately activated and generate a fast Na^+^ inward current, through the membrane, after membrane depolarization [1]. In atrial and ventricular myocytes, the sodium current (*I_Na_*) is principally governed by cardiac voltage-gated sodium channel 1.5 (Na_v_1.5) with a tinny contribution of Na_V_1.8 [2]. The human Na_v_1.5 channel is composed of a pore-forming α-subunit (227-kDa) and one or more auxiliary β-subunit (30-kDa) [3]. *SCN5A* gene with 80 kb length is located on chromosome 3p21 and consists of 28 exons which encode a protein of 2016 amino acid, the α-subunit of Na_v_1.5 channel [4]. This protein contains four homologous sites (DI–DIV), each composed of six transmembrane segments organized into two functional modules. Segments from one to four (S1–S4) generate the voltage-sensing module (VS), and segments five and six (S5–S6) jointly with P-loop create the pore module (PM). Finally, we can find an α-helical S4–S5 linker, whose function is to bind these two structures, the voltage-sensing and the pore modules. Moreover, there are intracellular linkers that are in charge of DI–DII, DII–DIII, and DIII–DIV binding, and more concretely, the DIII–DIV linker is the controller of pore closing, acting as a fast inactivation gate [5]. The VS and PM modules of the Na_v_1.5 constitute preferred therapeutic targets for the treatment of several cardiac sodium channelopathies. Particularly, flecainide, as well as other class IC antiarrhythmic drugs, bind to the central cavity of the pore and block sodium permeation directly [5]. The class IA antiarrhythmic drugs (e.g., procainamide) and the class IB antiarrhythmic drugs (e.g., lidocaine) might act on a smaller surface of the central cavity of the pore as well [5]. However, polyunsaturated fatty acids (PUFAs) and PUFA analogs have been shown to be antiarrhythmic by inhibiting Na_v_1.5 channel currents, probably through acting on the voltage-sensing S4 segments that control inactivation in these channels [6,7].

Since *SCN5A* is transcribed to mRNA until Na_v_1.5 is assembled into the plasma membrane to exert its function, there are several steps with different proteins involved. *SCN5A* mRNA is translocated from the nucleus to the cytoplasm by the Nucleoporin 107 (Nup107) protein. It has been demonstrated that Nup107 is increased whenever a hypoxic and oxidative situation has a place in the heart tissue [8]. Once in the cytoplasm, *SCN5A* mRNA is translated to protein and assembled to β1–β4 subunits in the rough endoplasmic reticulum (ER), and subsequently, Na_v_1.5 protein is exported to the Golgi by the nuclear import protein, RAN guanine nucleotide release factor (MOG1), and Protein Kinase A [9,10,11]. It has been previously reported that the defect of MOG1-Na_v_1.5 interaction causes Brugada syndrome [9]. After protein glycosylation in Golgi, Na_v_1.5 is ready for anchoring into the intercalated discs (ID) for the AP transmission between cardiomyocytes, being responsible for the electro-mechanical coupling through gap junctions, adherens junctions, and desmosomes [12,13]. Moreover, AP is conducted through the lateral membrane (LM) due to localized Na_v_1.5 channels into T-tubules and in the focal adhesion complex or costameres that link adjacent myocytes in the myocardium through extracellular matrix interaction [12,13]. This process is characterized by the presence of specific proteins that control Na_v_1.5 trafficking and anchoring [13].

At the beginning of cardiac AP, the cell membrane is depolarized, and Na_v_1.5 channels are activated due to positively charged arginine or lysine residues in S4 segments. All four charged S4 segments change their position in the cell membrane with an outward movement, which leads to the opening of the channel pore, conducting to an inward Na^+^ current [14]. Few milliseconds later, the depolarization process generates “fast inactivation”, where the Na_v_1.5 channel is closed and channel opening does not occur, due to an outward movement of S4 segments in domain III and IV, again. This dual role of the S4 segment is tightly integrated [15,16,17,18,19,20]. After a prolonged depolarization, P-loop and S5–S6 linker change their position with reference to the membrane, and Na_v_1.5 channels enter in a “slow inactivation”, leading to the termination of the Na^+^ current flow [15,21]. 

At the end of its life cycle, Na_v_1.5 is degraded by proteasome and autophagic degradation pathways [11]. 

## 2. Genomic Regulation of the Cardiac Sodium Channel

### 2.1. Genetic Code of SCN5A

Na_v_1.5 channel expression and function may be impaired due to variations in the genomic sequence of *SCN5A*, including missense, nonsense, splice-altering, and frame shift truncation [22,23]. These variations cause different cardiac diseases because of a loss- or gain-of-function and occasionally both, generating overlapped phenotypes [24]. For example, Brugada Syndrome (BrS) [24,25,26], progressive cardiac conduction disease (Lev-Lenegre disease) [27,28], and sick sinus syndrome [29,30] are some diseases caused by loss-of-function mutations in *SCN5A*. However, long QT syndrome type 3 (LQTS3) [24,31] and multifocal ectopic Purkinje-related premature contractions (MEPPC) [32,33,34] are due to gain-of-function mutations in *SCN5A*. Finally, a combination of gain- and loss-of-function mutations are associated with atrial fibrillation (AF) [35,36] and dilated cardiomyopathy (DCM) [34,36,37,38]. Some of these channelopathies are widely described in Section 4 of this review. Additionally, several *SCN5A* missenses can generate dominant-negative variants affecting Na_v_1.5 trafficking or gating at the cell surface [39,40,41]. A recent study demonstrated that most of *SCN5A* missense that generate loss-of-function variants exert a dominant-negative effect that confers a high burden of BrS [42]. Finally, post-translational modifications affecting Na_v_1.5 have an impact on the use of antiarrhythmic drugs, making these data quite relevant for future drug design [43].

### 2.2. Regulation of SCN5A Transcription

#### 2.2.1. Epigenetic Regulation of *SCN5A*

##### Regulation of *SCN5A* by Distinct Regulatory Elements and Histones

Gene transcriptional activation is not only modulated by transcription factors; in this process, the role of distinct regulatory elements (RE) is also important, as well as how these REs interact with chromatin, depending on DNA accessibility. Several authors have identified different roles of an enhancer cluster in the *SCN5A*-*SCN10A* locus, which modulate *SCN5A* gene expression [44,45,46]. RE-1 and RE-5 are located in an *SCN10A* and *SCN5A* intron, respectively, and RE-6, located downstream of *SCN5A*, contains genetic variants associated with PR intervals and QRS duration [44,45,47,48]. Moreover, Christoffels’ lab [49] has recently demonstrated that there are several downstream *SCN5A* REs acting as cardiac-specific “super enhancers”, concretely the intergenic region composed by RE6-9, which possess an extensive association with Histone H3 lysine (K) 27 acetylation (H3K27ac) [50]. RE6-9 has the ability to fine-tune *Scn5a*-*Scn10a* chromatin architecture modulating *Scn5a* expression. In addition, it has been identified that some single-nucleotide polymorphisms (SNPs) located in an enhancer region are able to regulate transcription factor binding and modulate gene expression. In particular, major alleles of rs6801957 and rs10428132 lead to *SCN5A* gene expression, while minor alleles cannot due to a loss of a T-box protein binding site [51,52]. Furthermore, an enrichment of H3K27ac and Histone H3 lysine (K) 4 trimethylation (H3K4me3) near of *SCN5A* promoter region in striated muscles regulates normal expression of *Scn5a* and improve the re-expression of *SCN5A* in denervated muscle [49,53,54]. Moreover, Lamin A/C (encoded by LMNA) is a component of the nuclear lamina, and its K219T mutation has been described to trigger a change in the distribution of the histone marks. Concretely, H3K9me and H3K27me, which are transcriptional repressive histone marks, and H3K4me3, which acts as transcriptional active histone mark, generate cardiac conduction defects through *SCN5A* inhibition and reduced *I_Na_* density [55].

##### Regulation of *SCN5A* by Transcription Factors

**Figure 1:**
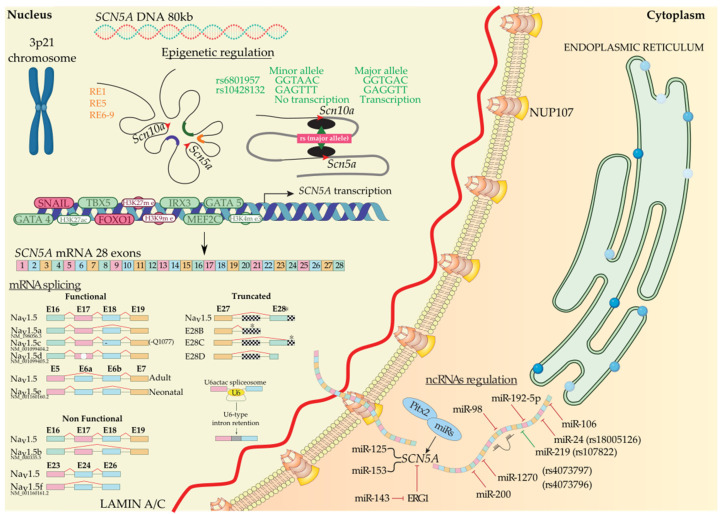
*SCN5A* biosynthesis: chromosomal localization, gene transcriptional activation modulated by regulatory elements, transcription factors, histones, and SNPs. Functional, non-functional, and truncated isoforms derived from mRNA splicing, mechanism of the U6-type intron retention, and post-transcriptional regulation mediated by ncRNAs. Alternative exon sequences, intronic or exonic sequences outside the open reading frame (squared), and stop codons (asterisks) are indicated.

During biosynthesis, *SCN5A* transcription is regulated by several transcription factors. Sometimes this transcription step can be enhanced or decreased, i.e., TBX5 has a binding site downstream of the *SCN5A* gene, and several authors have demonstrated that TBX5 knockout presents a decreased density of Na_v_1.5 that leads to arrhythmias and eventually sudden cardiac death [56,57,58]. Additionally, GATA4 and GATA5 have their binding site in the *SCN5A* promoter and intron 1 region. These transcription factors activate the *SCN5A* gene in human left ventricles, whereas heterozygous mutants for GATA4^+^/^−^ show short PR intervals [59,60,61]. Moreover, MEF2C has its binding site in the *SCN5A* promoter region and enhances *SCN5A* transcription [62,63]. Finally, IRX3 gain-of-function upregulates *SCN5A* mRNA levels [64,65], whereas, on the contrary, FOXO1 and Snail negatively regulate *SCN5A* mRNA levels [66,67,68,69,70] (Figure 1).

#### 2.2.2. Post-Transcriptional Regulation of *SCN5A*

##### Regulation of *SCN5A* by Alternative Splicing

After transcription, precursor mRNA copes with splicing and post-transcriptional modification to generate mature mRNA and finally translation into protein. Alternative splicing generate multiple functional (Na_v_1.5a, Na_v_1.5d, Na_v_1.5e, and Na_v_1.5c) and non-functional (Na_v_1.5b, Na_v_1.5f, and truncated) Na_v_1.5 variants [11]. Na_v_1.5a isoform is characterized by the deletion of exon 18. This isoform is only present in small rodents and, compared with full-length Na_v_1.5, leads to altered electrophysiological kinetics properties. There is no evidence of Na_v_1.5a expression in human cardiac cells [11,71]. Another alternative spliced variant of *SCN5A* generates Na_v_1.5c isoform, which has been identified as the most abundant isoform in humans. Na_v_1.5c is characterized by a 5′-trinucleotide deletion in exon 18, concretely a CAG—Glu (Q) in 1077 position, affected by the splicing machinery and generating a Na_v_1.5 variant that contains 2015 polypeptides instead of 2016. It has been identified that the electrophysiological properties of Na_v_1.5 and Na_v_1.5c are indistinguishable [71,72]. Na_v_1.5d is another Na_v_1.5 variant, where 120 bp fragment is deleted from exon 17. This Na_v_1.5d isoform is present in the fetal and adult human heart and has altered channel kinetics due to a reduction of open channel probability [71,73,74]. Finally, the last functional Na_v_1.5 variant is Na_v_1.5e and is generated by alternative splicing on exon 6. It can be found Na_v_1.5e with 5′-exon 6 in neonatal (exon 6a) or 3′-exon 6 (exon 6b) in any adult mammalian heart [4,75]. Na_v_1.5e contains a K211 residue, instead of D211 residue in Na_v_1.5, being responsible for slower kinetics of the channel [76]. Na_v_d1.5b is a non-functional Na_v_1.5 variant and is generated by the deletion of exon 17 and exon 18. Heterologous expression reveals that exon 17 encodes an essential Na_v_1.5 region that confers functionality to the channel [74,77]. This splice variant is present in mouse hearts, but there is no evidence of this variant in other mammals’ hearts [74]. On the other hand, deletion of exon 24 of Na_v_1.5 generates Na_v_1.5f variant; this isoform is highly detected in rat heart and human brain but not in the human heart [78,79]. Electrophysiological experiments evidenced that Na_v_1.5f is a non-functional variant [71]. Finally, it has been identified three C-terminal truncated spliced variants, E28B, E28C, and E28D, that generate reduced protein levels and no functional Na^+^ currents in the normal fetal and adult human heart [80]. In another layer of complexity, in a very recent study, it has been evidenced that minor introns modulate gene families at a post-transcriptional level. Concretely, U6actac, which is a minor spliceosome component, modulates Na_v_1.5 and Ca_v_1.2 protein levels through the removal of minor introns in *Scn5a* and *Cacna1c*, regulating electrophysiological properties of cardiomyocytes [81].

##### Regulation of *SCN5A* by Non-Coding RNAs

*SCN5A* mRNA degradation or translational repression can be mediated by microRNAs. It has been widely demonstrated that microRNAs exert their function controlling the development of several cardiac arrhythmogenic diseases; however, there is not much information about the *SCN5A*/Na_v_1.5 post-transcriptional control by microRNAs despite being an important modulator of cardiac arrhythmias [82,83,84,85]. A few years ago, data from our laboratory demonstrated that miR-98, miR-106, miR-200, and miR-219 directly regulated human *SCN5A*, while miR-125 and miR-153 regulate it indirectly, being evidenced that miR219 and miR200 modulate *Scn5a* expression in an opposite way in HL1 cardiomyocytes. Concretely, miR-200 decreases, while miR-219 increases *Scn5a* expression. Moreover, miR-219 is able to increase Na_v_1.5 protein levels leading to the subsequent rise in *I_Na_* [86]. In another study, we elucidated a complex regulatory network where Pitx2 transcription factor and microRNAs are involved, being able to modulate ion channel expression in atrial fibrillation [87,88,89]. Additionally, Zhao et al. [90] demonstrated that miR-192-5p exerts its function, reducing Na_v_1.5 expression and *I_Na_* density in humans. It has also been evidenced that *SCN5A* expression can be indirectly modulated through ERG1 mediated regulation by miR-143 [91]. Furthermore, a miR-24 binding site is generated in the coding sequence of *SCN5A* by the SNP rs1805126, decreasing *SCN5A* expression and *I_Na_* density [92]. Finally, it has been evidenced that some SNPs can generate new miRNAs binding sites at the *SCN5A* 3′UTR region. Previous work in our lab demonstrated that rs4073797 and rs4073796 polymorphisms create a new miR-1270 binding site in *SCN5A* 3′UTR, which imbalance *Scn5a* expression. Moreover, rs107822C localized upstream of miR-219a precursor impairs miR-219a expression deregulating *SCN5A*/Na_v_1.5 levels [93]. Although *I_Na_* is the most relevant during cardiac AP, the functional role of microRNAs controlling *SCN5A* expression is poorly described. Quite recently, a new class of RNAs has emerged, i.e., long non-coding RNAs (lncRNAs), which are long RNA molecules with >200 nt length and scarce protein-coding properties, but being able to modulate several biological processes and thus participating in pathogenesis [94,95]. It has been demonstrated that some lncRNAs modulate cardiac AP regulating the slow component of the delayed rectifier potassium current (*I_KS_*) or transient outward potassium current (*I_to_*), concretely, lncRNA-Kcna2as and lncRNA-MALAT1, respectively [96,97], and more recently, it has been elucidated that lncDACH1 modulate Na_v_1.5 protein distribution through dystrophin binding which determines Na_v_1.5 membrane anchoring [98]. Studies about how cardiac AP can be modulated by lncRNA need to be further developed, as these molecular mechanisms could reveal a potential therapeutic work line. 

## 3. Non-Genomic Regulation of the Cardiac Sodium Channel

Being an ion channel, Na_v_1.5 is first synthesized as a primary protein chain that is subsequently folded in order to acquire the pore-forming three-dimensional conformation [11]. This tertiary structure is then assembled with its beta subunits, most likely (β1), and trafficked through the Golgi apparatus to be targeted to the corresponding cell membrane compartments [99]. Along this whole process, Na_v_1.5 went through distinct non-genomic regulatory modifications and quality control steps conferring its unique conformational and functional identity as a voltage-gated sodium channel [11,100]. These steps are ensured by a growing set of regulatory proteins that have been demonstrated to covalently or non-covalently interact with Na_v_1.5 [101]. In addition to the interacting proteins, Na_v_1.5 function has been demonstrated to be influenced by wider intracellular (oxidative stress, metabolic stress, electrolyte homeostasis, etc.) and extracellular (pH, temperature, hormones, etc.) factors. All these factors are discussed subsequently in this review.

### 3.1. Regulation of Na_v_1.5 Biosynthesis and Post-Translational Modifications

#### 3.1.1. Regulation of Na_v_1.5 Translation and ER Retention

**Figure 2:**
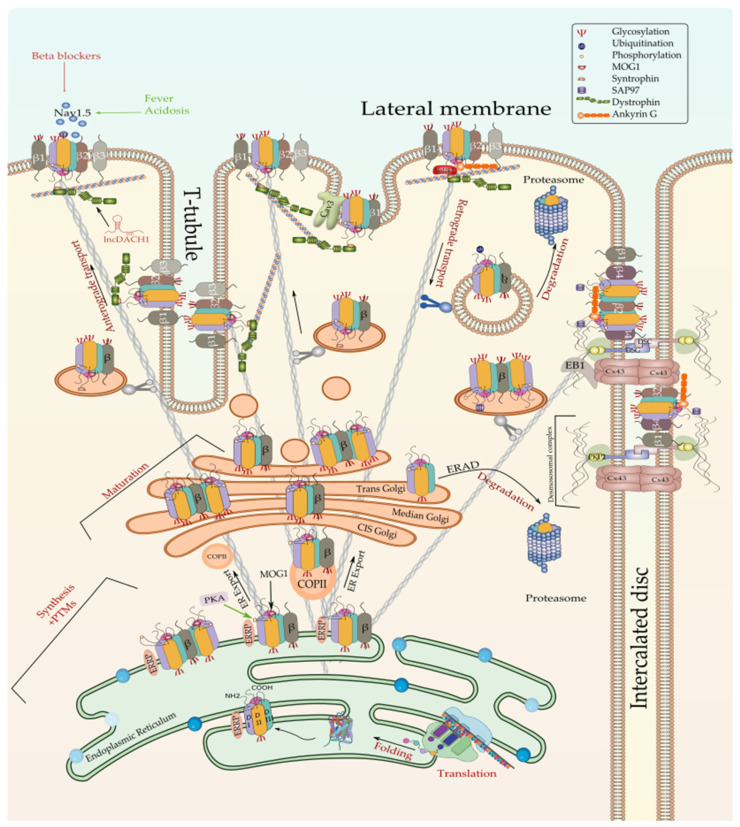
Biosynthesis and degradation pathways of Na_v_1.5. Only one of the possible scenarios where Na_v_1.5 assembles with one or more β-subunits early at the ER is depicted here. Furthermore, one possible scenario where ERAD-dependent degradation exclusively affects α subunit rather than α-β assembly is shown here since no information is currently available about the detailed process. ERRP—endoplasmic reticulum retention protein; β—beta subunit; PKA—protein kinase A; MOG1—RAN guanine nucleotide release factor; COPII—coat protein complex II; ERAD—ER-associated degradation; Cx43—connexin 43; PKP2—plakophilin 2; DSG—desmoglein; DSC—desmocollin; EB1—end-binding 1; Cav3—caveolin 3; PTMs—post-translational modifications.

The translation of Na_v_1.5 starts in the cytosol and then pursue into the endoplasmic reticulum (ER). Anchoring the ribosome with the elongating Na_v_1.5 polypeptide chain to the ER occurs when a signal peptide is recognized by the signal recognition particle (SRP) that targets the active ribosome to the rough endoplasmic reticulum (ER) membrane. Unlike cytosolic proteins, which have their signal peptide generally within the amino terminal, ion channels contain numerous signal sequences that are not restricted to the amino terminal [102]. Although the signal sequences of some ion channels such as Kv1.3 and CFTR have been already mapped to the second transmembrane spanning domain, almost 200 amino acids downstream from the NH_2_ terminal [102], that of Na_v_1.5 are not yet identified. Once anchored, the ribosome translocates the elongating polypeptide chain into the ER lumen [103]. As a transmembrane protein, the nascent Na_v_1.5 is soon pushed to the ER membrane, where it is anchored and retained [103]. The ER retention is thought to occur when specific ER retention motifs embedded in the elongating Na_v_1.5 polypeptide (most likely in the DI-DII linker of the sodium channel [104]) binds a cytosolic signal recognition particle (endoplasmic reticulum recognition particle, ERRP), that then directs the Na_v_1.5-ERRP complex to receptors within the ER membrane [105,106,107]. The complex Na_v_1.5-ERRP is then trapped within the ER, ensuring that the newly formed channel does not leave the ER membrane before finishing the folding and assembly steps [102]. At this level, several regulatory proteins and residues are reported to bind to this complex and facilitate the folding and maturation of the nascent protein [11,102,105,106,107,108,109,110] (Figure 2).

The correct folding of the newly synthesized Na_v_1.5 channels is commonly thought to be a condition for their forward trafficking to the cell membrane and their proper gating function. This notion has been tested by the exploration of Na_v_1.5 trafficking-deficient mutants such as R282H, A124D, and V1378M that, due to folding defects, they failed to exit the ER and thus to reach the cell membrane [111,112]. Although the importance of this step in the life cycle of any ion channel, very scarce information is currently available about the mechanism of Na_v_1.5 folding and its regulation. Nonetheless, it is currently established that one of the prerequisites for proper Na_v_1.5 folding is core-glycosylation, as will be discussed later in this review [113]. In addition, molecular chaperone proteins such as protein disulfide isomerases (PDI), ER oxidoreductases (ERO), 70 kDa heat shock proteins (Hsp70), 90 kDa heat shock proteins (Hsp90), as well as calnexin and calreticulin, have been demonstrated to regulate the folding of the nascent proteins and the ER-associated degradation of the misfolded proteins [114,115,116,117,118]. However, their implication in the Na_v_1.5 folding process has not been specifically studied yet. Interestingly, Na_v_1.5 H558R polymorphism has been shown to have a corrective effect on the misfolded R282H mutant by restoring its trafficking to the cell membrane and thus limiting the misfolding of the mutant through a physical interaction [111,119].

Some antiarrhythmic drugs such as mexiletine, quinidine, and flecainide proved their efficiency rescuing the trafficking of some misfolded Na_v_1.5 variants, thus playing the role of pharmacological chaperones [112,120]. In addition, curcumin, a major constituent of turmeric known to block the ER calcium pump, has also been reported as effective in rescuing the *I_Na_* current of L325R misfolded Na_v_1.5 channels [40]. Low temperature has also been demonstrated to trigger the rescue of misfolded Na_v_1.5 mutants [120], probably through slowing the folding process, which prevents protein misfolding and aggregation [121]. 

#### 3.1.2. Co-Translational and Post-Translational Regulation of Na_v_1.5

##### N-Linked Glycosylation of Nascent Na_v_1.5

One of the earliest modifications that the Na_v_1.5 undergoes co-translationally once inserted into the ER is the N-glycosylation [99,113]. This quality control step has been first evidenced in the rat heart by Cohen and Levitt, who have found that glycosylation increases Na_v_1.5 mass by only 5%, compared to 25–30% increases observed in other voltage-gated sodium channel isoforms [122]. Glycosylation initiates in the ER and terminates in the Golgi [113,123]. In the ER, glycosylation initiates when glycan (Glc3Man9GlcNAc2) is dissociated from a lipid derivative by oligosaccharyl transferase (OST) and bind to the amide nitrogen of asparagine (N) localized in the extracellular side of the nascent Na_v_1.5 protein [100,124]. Although no validated “map” of the N-glycosylation sites has been published yet for Na_v_1.5, 13 potential external N-glycosylation sites have been identified in human Na_v_1.5 [125], and at least 14 putative N-linked glycosylation sites have been reported in the rat cardiac sodium channel [122]. The N-glycosylation of the newly formed cardiac sodium channel has been reported to be a prerequisite for proper Na_v_1.5 folding and subsequent surface expression as well as an assembly with its β subunits [99,113,126]. According to Arakel et al., Na_v_1.5 maturation strongly depends on the presence of the auxiliary β1 that binds to the pore-forming α subunit and promotes its glycosylation and its trafficking to the cell membrane [127]. 

In this context, N-glycosylated Na_v_1.5 is thought to undergo subsequent serial de-glucosylation steps and extreme quality controls involving the ER-resident chaperones, which will ensure that only correctly folded and fully glycosylated channels can be trafficked [113,123,128]. Interestingly, Mercier et al. found that early N-glycosylated Na_v_1.5 channels generated in the ER could reach the cell membrane through an unconventional trafficking pathway bypassing the Golgi stacks while functional channels are trafficked through the conventional pathway that is Golgi-dependent [113]. In addition, ER-resident chaperones such as Calnexin and Calreticulin have been reported to play a crucial role in controlling ion channels folding and efficient export to the Golgi [129,130,131]. However, there is no evidence of physical interaction of Calnexin and Na_v_1.5 despite their proven co-localization in the ER [132,133]. While properly folded Na_v_1.5 are trafficked forward to the cis-Golgi where they will be fully maturated, misfolded Na_v_1.5 are retained in the ER to be later degraded, most likely through the activation of the unfolded protein response (UPR) pathway and/or ER-associated degradation (ERAD) pathway that is linked to the cytoplasmic ubiquitin-proteasome pathway [134,135,136]. 

##### Phosphorylation and Dephosphorylation of Na_v_1.5

In addition to N-linked glycosylation, Na_v_1.5 undergoes phosphorylation as a post-translational modification [137]. Thirty years ago, Shubert et al. brought the first evidence of Na_v_1.5 phosphorylation by protein kinase A (PKA) through the activation of the β-adrenergic system by isoproterenol, which led to an increased level of cAMP, which in turn reduced Na^+^ current (*I_Na_*) [138]. These findings were further confirmed by a subsequent study by Frohnwieser and his co-worker, who showed that combined cytosolic injection of cAMP and a PKA activator increased *I_Na_* suggesting a modulatory effect of PKA on human Na_v_1.5 [139]. The same study demonstrated that this modulatory effect of PKA is conferred by the DI–DII intracellular linker of Na_v_1.5. In this regard, it has been reported that the rat Na_v_1.5 protein sequence harbors two distinct sites for PKA phosphorylation that were mapped to serine positions S526 (525 in human) and S529 (528 in human) [100,140,141,142]. These sites are localized in the cytosolic loop interconnecting DI and DII of Na_v_1.5, where the three putative RXR-type (R479KR481, R533RR535, and R659QR661) ER retention motifs have been localized too [104,137,143]. Zhou et al. have previously demonstrated that PKA activation promotes trafficking of channels to the plasma membrane [143]. In the same context, Scott et al. have shown that a PKA-PKC mediated phosphorylation of NMDA receptor masks its ER retention motifs leading thus to its release from the ER and exportation to the cell membrane [144]. Taken together, these findings suggest a similar mechanism where the phosphorylation of Na_v_1.5 at S525 and S528 by PKA leads to changes in the Na_v_1.5 conformation that masks the ER retention signals and eases the trafficking of the channel to the cell membrane [142,145]. This is consistent with the idea that proper folding of Na_v_1.5 unmasks its ER retention motifs and facilitates its forward trafficking to the Golgi apparatus [106].

In an antagonistic way to PKA, Na_v_1.5 is downregulated by protein kinase C (PKC)-mediated phosphorylation which leads to a reduced channel density at the cell surface and *I_Na_* decay [146]. Although ten different PKC isoforms have been identified in human ventricular myocytes and in different animal species [147], isoform-specific activation/inhibition studies suggested εPKC isoform as the key player in the PKC-mediated regulation of Na_v_1.5 and *I_Na_* [148,149]. Nonetheless, PKCδ-mediated Na_v_1.5/*I*_Na_ downregulation either directly through phosphorylation at S1503 or indirectly through elevated mitoROS production has been reported [150]. In addition, a minor role of αPKC reducing *I_Na_* through angiotensin II has also been described [151]. As a direct mechanism, the PKC (particularly εPKC) effect on Na_v_1.5 and *I_Na_* has been partially attributed to the phosphorylation of a conserved serine S1503 of the DIII-DIV cytosolic linker of Na_v_1.5 [152,153]. However, intracellular metabolic changes have been described as a mediator of PKC activation and PKC-mediated phosphorylation of Na_v_1.5 [150]. In this regard, high intracellular levels of NADH have been described as triggers of PKC, thus leading to overproduction of mitochondrial reactive oxygen species (mitoROS) and *I_Na_* decay [154,155,156]. This effect has been demonstrated to be mediated by glycerol 3-phosphate dehydrogenase 1 (GPD1L) [157] and could be reversed by NAD^+^-mediated PKA activation [154,158,159,160]. Interestingly, Fouda et al. have demonstrated that PKA and PKC phosphorylation pathways could be activated by Cannabidiol and Estradiol and that this activation could rescue the high glucose-induced changes in Na_v_1.5 properties [161,162].

Importantly, not far from the PKA phosphorylation sites in Na_v_1.5 DI–DII linker, there is a Ca^2^^+^/Calmodulin-dependent Protein Kinase II (CaMKII) phosphorylation site as well, which was mapped to S516 [163]. This CaMKII phosphorylation site is not the only one in Na_v_1.5 since Ashpole et al. have identified four extra potential sites; all of them are localized in DI–DII linker, suggesting linker I as a hotspot for Na_v_1.5 phosphorylation [163]. However, a recent study by Herren et al. identified 23 sites along Na_v_1.5 intracellular regions that could be phosphorylated by CaMKII in human Na_v_1.5 [164]. More recently, Burel et al. identified two further CaMKII phosphorylation sites localized in the C-terminal region of Na_v_1.5 [165]. Several studies have shown that Na_v_1.5 is regulated by CaMKII and that activation of this kinase increases the so-called pathogenic late cardiac sodium current *I_NaL_* [166]. Interestingly, El Refaey et al. demonstrated that *I_NaL_* could also be regulated by B56α, the key regulatory subunit of the PP (protein phosphatase) 2A holoenzyme [167]. This phosphatase is targeted by ankyrin-G to the Na_v_1.5-CaMKII -βIV spectrin axis at the ID where it is thought to dephosphorylate Na_v_1.5 at S571 in the DI-DII linker via B56α balancing, thus the CaMKII-dependent phosphorylation of the cardiac sodium channel. According to a study by Deschênes et al., inhibition of CaMKII slowed Na_v_1.5 channel current decay, produced a depolarizing shift in fast inactivation, and slowed entry into inactivated states [168].

Na_v_1.5 is also phosphorylated by Tyrosine kinases. In this regard, phosphorylation of Na_v_1.5 by the Src family Tyrosine kinase Fyn has been first reported by Ahern and co-workers, who have demonstrated that this kinase acts by increasing the rates of recovery from fast-inactivated states, thus impairing the steady-state inactivation of Na_v_1.5 [169]. Fyn kinase acts most likely on Tyr1495 of Na_v_1.5 not far from the Ile-Phe-Met (IFM) motif of DIII–DIV linker that is known to modulate the rapid inactivation process of the channel [5]. In the heart, Fyn tyrosine kinases are reported to co-localize with Na_v_1.5 channels at adherens junctions, where they modulate electrical coupling and propagation of action potential [170,171]. Iqbal et al. found that the major Na_v_1.5 splice variants Q1077 and delQ1077 are differentially phosphorylated by Fyn kinase, which results in coordinated steady-state rapid inactivation kinetics for smooth electrical activity of the heart [172]. The same researchers suggested a multistep mechanism by which Fyn kinases bind and modulate Na_v_1.5. This mechanism starts by the association of Fyn kinase to proline-rich regions in the DI–DII linker and C-terminal region of Na_v_1.5, which activates the phosphorylation of neighboring tyrosine residues in the N-terminal region (Y68, Y87, and Y112), DIII–DIV linker (Y1494, Y1495), and C-terminal region (Y1811, Y1889) [169,172,173]. Particularly, Y1494 and Y1495 of the DIII–IV linker have been demonstrated to play an essential role in the anchoring of Ca2^+^/Calmodulin to the Na_v_1.5 inactivation gate, and thus Fyn-mediated phosphorylation of the two Tyrosine residues has been suggested to reduce or abolish calmodulin binding and to impair the interaction of the side chain with the inactivation gate receptor [174]. 

Additionally, Na_v_1.5 has been reported to be dephosphorylated by the protein tyrosine phosphatase 1 (PTPH1), which interacts with the Na_v_1.5 PDZ domain binding site at the C-terminal region [101]. PTPH1–mediated dephosphorylation of Na_v_1.5 modulates its gating by shifting the steady-state inactivation towards hyperpolarized potentials [175].

##### Arginine Methylation

Beltran-Alvarez and co-workers evidenced for the first time that Na_v_1.5 is post-translationally modified by arginine methylation at three residues (R513, R526, and R680) within the Na_v_1.5 DI–DII linker [176]. This modification is catalyzed by arginine methyltransferases (PRMT) PRMT-3 and PMRT-5 and leads to an increased expression of Na_v_1.5 in cell surface [177]. Studying the PTMs of Na_v_1.5 in end-stage heart failure patients, the same team demonstrated that methylation of R526 is the major quality control step of any Na_v_1.5 arginine or lysine residue [178].

##### N-Terminal and Lysine Acetylation

Another PTM during the Na_v_1.5 life cycle is the acetylation process. Two types of acetylation have been reported so far: reversible and irreversible. The first type is mediated by histone acetyltransferases (HATs) which exert N-terminal acetylation of a Na_v_1.5 lysine residue leading to enhanced trafficking of Na_v_1.5 and therefore to an increased *I_Na_* current [179], whereas the second type of acetylation is mediated by N-terminal acetyltransferases (NATs), where a Na_v_1.5 alanine residue is acetylated and has been reported as a Na_v_1.5 degradation signal [177]. Interestingly, native Na_v_1.5 channels purified from end-stage heart failure patients were reported to lack the initiation of methionine and be acetylated at the resulting initial alanine residue [178]. Recently, Vikram et al. showed that Na_v_1.5 undergoes reversible lysine acetylation. For instance, sirtuin 1 deacetylase (Sirt1), an NAD^+^-dependent lysine deacetylase, has been demonstrated to regulate Na_v_1.5 channels by deacetylating lysine residue 1479 (K1479) in the DIII–DIV linker, which promotes Na_v_1.5 cell surface expression and increases *I_Na_* [180]. Interestingly, the murine model of cardiac Sirt1 deficiency presents fatal cardiac conduction defects as a result of K1479 hyperacetylation, which decreases Na_v_1.5 cell surface expression and reduces *I_Na_*. These arrhythmogenic substrates are similar to those characterizing human Na_v_1.5 loss-of-function cardiac arrhythmias suggesting that Na_v_1.5 Sirt1-mediated deacetylation is crucial for the proper function of the cardiac sodium channel. It is noteworthy that the authors of this study raised an interesting point regarding the role of the functional interaction and interplay between different PTMs fine-tune regulating the Na_v_1.5 channel expression and function. In this regard, it has been suggested that Na_v_1.5 is regulated by Sirt1-mediated interaction between lysine acetylation and the ubiquitination in one hand and NAD^+^ dependent interplay between PKC-mediated phosphorylation and Sirt1-mediated deacetylation in another hand [180]. 

##### SUMOylation

Although more than 25 years have passed since the discovery of SUMOylation, a post-translational modification conjugating a small ubiquitin-like modifier (SUMO) molecule to a lysine residue in the substrate protein [181], very scarce information are currently available about the regulation of Na_v_1.5 by SUMOylation. For instance, only one study, that of Plant et al., has reported that one of the mechanisms underlying *I_NaL_* elevation in response to acute cardiac hypoxia is the quick SUMOylation of Na_v_1.5 channels at the cell surface [182]. Particularly, SUMOylation of K442 residue has been reported to contribute to the pathological increasing of I_NaL_ and action potential prolongation through activation of Na_v_1.5 channels when they should normally be inactivated. 

##### S-Nitrosylation

S-nitrosylation, a PTM consisting of the covalent binding of a nitrogen monoxide (NO) moiety to the thiol side chain of cysteine in the target protein, has recently gained progressive attention as a crucial quality control step that is required for the proper function of a given protein [183]. In the cardiomyocytes, NO is produced by neuronal nitric oxide synthase (nNOS) [184]. nNOS mediated S-nitrosylation of Na_v_1.5 has been demonstrated to maintain *I_NaL_* [185]. Interestingly, nNOS has been shown to interact with Na_v_1.5 via its regulating protein α1-syntrophin, which acts as a scaffolding protein bringing together Na_v_1.5 with nNOS and plasma membrane Ca-ATPase (PMCA4b) (an inhibitor of nNOS activity) [186]. Therefore, LQTS-associated α1-syntrophin mutation has been demonstrated to break the SNTA1- PMCA4b association neutralizing, thus the nNOS inhibition and increasing Na_v_1.5 S-nitrosylation, which in turn increase *I_NaL_* currents [186]_._ A similar effect has been observed with a decreased caveolin 3(Cav3) expression, which has been shown to enhance S-nitrosylation of Na_v_1.5 through increasing the nNOS activity, which increased *I_NaL_* in cardiomyocytes [187]. However, a very recent study by Wang and co-workers suggested an indirect mechanism by which S-nitrosylation modulates the cardiac sodium channel expression and function. For instance, NO has been demonstrated to down-regulate *SCN5A* expression and Na_v_1.5 function through S-nitrosylation of regulatory transcription factor FOXO1 [188]. These findings increase our current understanding of the role of redox and free radicals in the regulation of Na_v_1.5 function (see [100] for further review).

##### Lipoxidation

Lipoxidation refers to the establishment of covalent adducts between reactive products of lipid peroxidation and macromolecules such as proteins, phospholipids, and DNA [189]. Recently, lipoxidation gained interest as a post-translational modification of the cardiac sodium channel that gives further evidence on the regulation of Na_v_1.5 by oxidative stress [190]. Nonetheless, little information is currently available about the mechanism of Na_v_1.5 regulation by lipoxidation. In this respect, in vitro data by Nakajima and co-worker provided the first evidence that Na_v_1.5 is post-translationally modified by lipoxidation during oxidant injury and that sodium channel dysfunction evoked by lipid peroxidation could be prevented by scavenging Isoketals (IsoKs), which are the most reactive products of lipoxidation [191]. 

##### Methionine Oxidation

A previous study by Quiñonez et al. demonstrated that skeletal Na_v_1.4 fast inactivation could be impaired by oxidizing at least two methionine residues in the channel [192]. These findings have been supported in cardiac Na_v_1.5 as well, where oxidative modification of the methionine within the IFM motif has been shown to lead to a drastic loss of Na_v_1.5 inactivation [193]. Interestingly, Na_v_1.5 channels and *I_Na_* currents have been reported to be indirectly modulated by CaMKII, the activation of which depends on the oxidation of its own methionine residues [194].

##### Palmitoylation

Palmitoylation (also called S-acylation) is the PTM of protein cysteines with saturated fatty acids that modify protein hydrophobicity and thereby influence their function [195]. Palmitoylation has been reported to regulate ion channel’s function, most likely through controlling their trafficking and cell membrane expression [99,196]. An early study by Schmidt et al. showed that Na_v_1.5 is subject to palmitoylation [99]. However, palmitoylation has been demonstrated to slightly influence cell surface expression of Na_v_1.5 and rather significantly impact channel availability by regulating the voltage dependence of steady-state inactivation in both HEK293 cells and cardiomyocytes [197]. Additionally, cysteine residues predicted to be palmitoylated in Na_v_1.5 are mapped to the DII–DIII linker of the channel by prediction algorithms [197]. 

#### 3.1.3. Regulation of the ER-to-Golgi Trafficking

Well folded and assembled proteins are supposed to cross the ER-Golgi space in vesicle budding guided by cytoskeletal proteins [198]. Studying the subcellular distribution of the cardiac sodium channel Na_v_1.5 in HEK293 Cells and canine cardiac myocytes, Zimmer et al. noticed an accumulation of the intracellular channels within the ER and a lower channel density in the Golgi apparatus. Thereby, they proposed that ER plays the role of an intracellular reservoir where sodium channels are transiently stored [199]. As discussed previously, stimulation of PKA likely results in the activation of the ER-to-Golgi trafficking, which in turn leads to a rapid increase of the channel density in the cell membrane [104]. However, the whole mechanisms underlying the ER exit of Na_v_1.5 to the Golgi is not yet fully deciphered, and current advances in this topic show that not only the PKA-mediated phosphorylation of the Na_v_1.5 ER retention sites is what facilitates its ER-Golgi exportation. That is, several proteins and enzymes have been reported to bind to Na_v_1.5 once retained to the ER and enhance its release. In this context, Wu et al. have identified the Ran-guanine nucleotide release factor (RANGRF or MOG1) as a cofactor of Na_v_1.5, which by binding to its intracellular loop DII–DIII facilitates its cell surface expression [200]. Using the DII–DIII linker of Na_v_1.5, in yeast two-hybrid analyses, the team demonstrated that MOG1 is crucial for the optimal expression of Na_v_1.5 and promotes its ER export and intracellular trafficking to the plasma membrane [200]. These findings are consistent with Chakrabarti et al. study, which showed that silencing of MOG1 expression by small interfering RNAs caused retention of Na_v_1.5 in the ER, reduced Na_v_1.5 plasma membrane expression, and disrupted the Na_v_1.5 targeting to the cell surface, in particular, to the caveolin-enriched microdomains (caveolae) [201]. A subsequent mutational study performed by Yu et al. further revealed that mutations in the amino acids E83, D148, R150, and S151 of MOG1 disrupt its interaction with Na_v_1.5 and significantly reduce the cardiac sodium channel trafficking to the cell surface, suggesting that these amino acids are important for the MOG1-Na_v_1.5 binding and interaction [9]. The same team found that MOG1-mediated trafficking and function of Na_v_1.5 requires the interaction of MOG1 with two small GTPases SAR1A and SAR1B and that the knockdown of both enzymes abolishes the function of MOG1 [202]. Furthermore, it has been demonstrated that activation of SAR1 leads to the recruitment and internalization of Na_v_1.5 cargo into the coated transition vesicle COPII-coated vesicles that will ensure its ER-to-Golgi trafficking [202]. The Na_v_1.5 ER export is also controlled by Dynamitin as demonstrated by Chatin et al., who have proved, using a yeast two-hybrid system, that Dynamitin (C-terminal domain), interacted with the Na_v_1.5 DI–DII linker between amino acids 417 and 444 and that this interaction is crucial for the Na_v_1.5 cell-surface density probably through controlling the ER-to-Golgi trafficking [203]. 

#### 3.1.4. Regulation of Na_v_1.5 Maturation and Golgi Export

Once in the Golgi, N-glycosylated Na_v_1.5 undergoes additional mannose trimming and terminal glycosylation where acetyl-glucosamine, oligosaccharides, and finally sialic acid residues are sequentially added as the protein crosses the distinct Golgi cisternae. It has been demonstrated that glycosylation regulates voltage-gated sodium channels (including Na_v_1.5) gating, inactivation, and recovery process during cardiac AP by interfering with the electric field near the gating sensors [204,205,206,207,208]. Hence, it has been suggested that extracellular sialic acid residues, which are negatively charged at physiological pH, modulate the sensitivity of the Na_v_1.5 voltage sensor domains to the transmembrane electrical potential fluctuation [209]. Particularly, sialic acid residues localized to DI S5-S6 have been demonstrated to regulate the sialic acid-dependent gating of Na_v_1.5 [125].

Mature Na_v_1.5 (fully glycosylated) are exported from the Golgi apparatus, which acts as a major secretory sorting hub that targets newly synthesized proteins to their final subcellular destinations [210]. Although the current knowledge on the exact mechanisms regulating the Na_v_1.5 export from the Golgi and trafficking to the cell membrane is still limited, a recent study by Ponce-Balbuena and co-workers reported that Na_v_1.5 Golgi export is driven by a trafficking signal localized in its terminal COOH region. This signal corresponds to a binding site of the adaptor protein complex 1 (AP1) mapped to Na_v_1.5’s Y1810 residue. AP1-marked Na_v_1.5 will be then incorporated into clathrin-coated vesicles that will migrate to the cell membrane where the channel will be anchored [211]. The same team showed that the Na_v_1.5 cross the Golgi-cell membrane space by a common anterograde trafficking pathway as Kir2.1. These findings support previous studies demonstrating that both ion channels form a channelosome that shares common trafficking, targeting, anchoring, recycling, and degradation pathways [212,213].

#### 3.1.5. Regulation of the Na_v_1.5 Targeting to the Cell Membrane

**Figure 3:**
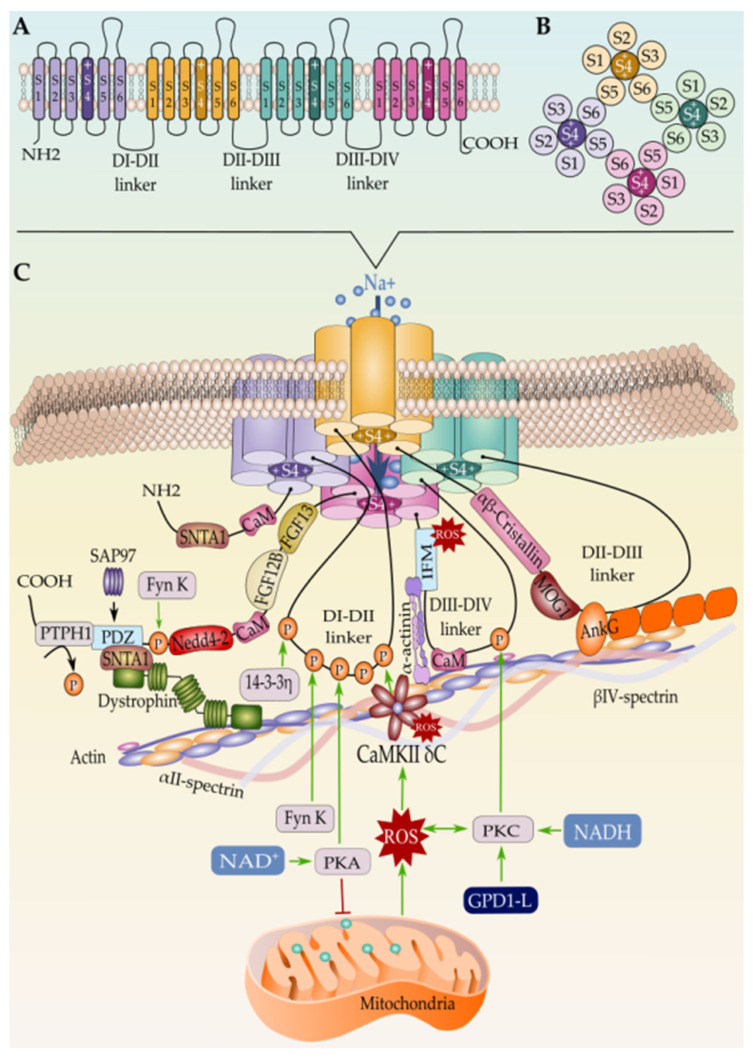
(**A**) Schematic representation of the Na_v_1.5 secondary structure, (**B**) the intracellular view of the channel, and (**C**) the tertiary structure along with the interacting proteins. Only proteins with known binding sites in Na_v_1.5 are represented here. Mechanism of Na_v_1.5 regulation by the mitochondrial reactive oxygen species ROS is represented as well.

Over the last few years, it became widely accepted that not all the Na_v_1.5 proteins synthesized in one cardiomyocyte undergo the same regulatory steps till reaching their final localization in the cell membrane [214]. After years of debate and controversial studies about the subcellular distribution of the cardiac sodium channel, the new cellular imaging techniques excluded the idea of an exclusive expression of Na_v_1.5 at the ID [171,215] and gave way to a more conceivable model that suggests a multi-pool aggregation of Na_v_1.5 along with the cellular membrane compartments including the LM and the T-tubules [216,217,218]. Being in one membrane domain or the other put the Na_v_1.5 in distinct microenvironments composed of different interacting proteins that regulate its gating function and biophysical properties. Above all these interacting proteins, beta subunits are without doubt the ones that most gained interest in this field over the last few decades as their presence and function are dependent on the presence of the pore-forming α-subunit (Figure 2 and Figure 3).

##### Regulation of Na_v_1.5 by β-Subunits

The β subunit family consists of four different proteins β1–4 encoded by four genes, *SCN1B–SCN4B*, respectively, with β1 alternatively spliced into two isoforms, β1A and β1B [219]. As mentioned earlier in this review, the β-subunits, most likely β1-subunits, assemble with Na_v_1.5 at the endoplasmic reticulum and influence its maturation and trafficking to the plasma membrane [127,220]. Alpha-beta subunits assembly is either covalent (β2 or β4) or non-covalent (β1 or β3) [221]. Particularly, β 4-Na_v_1.5 covalent association is ensured by an extracellular cysteine–cysteine single disulfide bond [222,223], while β2 does not form a disulfide linkage at this position with Na_v_1.5 as recently specified [5], whereas β1 and β3 non-covalently interact with Na_v_1.5 through the channels DIV and DIII voltage gating domain respectively [224]. 

Despite the structural similarities between β2/β4 on one hand and β1/β3 on the other hand, their expression differs from one cellular sub-domain to another. Inside the cardiomyocyte, β3 are expressed at the T-tubules and β4 at the ID, while β1 and β2 are found at both locations [215,225,226]. Zimmer et al. have suggested that, unlike β2, β1 associates to Na_v_1.5 early at the ER, and both α and β1 subunits are trafficked together to their final destination at the cell membrane [227]. Subsequent studies revealed that β1-subunits enhance the α-subunits dimerization and promote the dominant-negative effect of trafficking defective mutants [228]. β2 has been reported to promote surface localization of Na_v_1.5 [229]. Importantly, β3 subunits have been demonstrated to bind to Na_v_1.5 in multiple sites and promote the formation of α subunit oligomers, including trimers [230]. However, β4 has been reported as a modulator of Na_v_1.5 kinetic and gating properties by increasing *I_Na_* [231]. Taken together, these findings are consistent with the idea that the distinct sodium channel β subunits provide support for the pore-forming subunit, facilitate the trafficking of the mature channel to the different membrane domains, and modulate the gating function of Na_v_1.5 by increasing the *I_Na_* [232,233,234,235,236]. More details regarding the regulation of Na_v_1.5 by β subunits in the context of sodium channelopathies are discussed in Section 4 of this review. 

##### The Na_v_1.5 and the Intercalated Disc Interactome

As suggested by the Delmar research team, several evidence point to the fact that the ID is not a hub of proteins playing independent functions within the cardiomyocyte, but rather a network of molecules interacting together in order to fulfill a specific function (AP propagation, cell-to-cell coupling, cardiac excitability, etc.) that cannot be accomplished if this “interactome” is impaired [237]. As a component of the ID proteins, Na_v_1.5 has been demonstrated to be in the heart of this interactome by physically and functionally associating to several proteins belonging to this macromolecular complex. 

In this context, it is currently well known that Na_v_1.5 targeted to the ID are “tagged” with synapse-associated protein 97 (SAP97), a scaffolding MAGUK ((membrane-associated guanylate kinase) protein that is abundantly expressed in human and rat ventricular myocardium [238]. SAP97 has been introduced as the determinant of the Na_v_1.5 ID pool as it plays an important role in targeting Na_v_1.5 along with Kir2.1 to this cell membrane domain [238,239]. Both channels were structurally evidenced to co-assemble to SAP97 by their C-terminal domains [238,240]. For Na_v_1.5, it is assumed that the last three amino-acids (serine–isoleucine–valine or SIV motif) of the C-terminal region form a PDZ (postsynaptic density protein (PSD95), Drosophila disc large tumor suppressor (Dlg1), and zonula occludens-1 protein (zo-1) domain binding motif) that interacts with the syntrophin–dystrophin complex at the cardiomyocyte LM and PDZ domains of SAP97 at the ID [218]. In the absence of the PDZ-domain-binding motif of Na_v_1.5 or SAP97, Na_v_1.5 expression at the cell surface decreased, thus leading to a reduction in the cardiac *I_Na_* in vitro [241]. However, a subsequent study by the same team demonstrated that in vivo ablation of SAP97 did not change Na_v_1.5 localization and function, but it did decrease the cardiac potassium currents [242]. The authors of these studies justified this discrepancy by the fact that SAP97 silencing in vitro is induced in adult cardiomyocytes while in vivo, it is a constitutive ablation present early in development, which may impact protein expression and interactions.

In addition, the Na_v_1.5-SAP97-Kir2.1 complex has been demonstrated to reach the ID through the microtubule highway [133,238,239,243]. Although the exact mechanism by which Na_v_1.5 is targeted to the ID is not yet fully discovered, part of it is already elucidated. A few years ago, Agullo-Pascual et al. proved for the first time that the microtubule plus-end tracking protein “end-binding 1” (EB1) is captured to the IDs by connexin 43 (cx43), which facilitates the cargo delivery, including Na_v_1.5 [244]. These findings are consistent with Marchal and co-workers’ recent study in which they have further proved that EB1 modulates Na_v_1.5 trafficking to the IDs and that loss of EB1 function leads to reduced *I_Na_* and conduction slowing [245]. Moreover, EB1 has been previously demonstrated to bind directly to CLASP2 (cytoplasmic linker associated protein 2) and form a complex at the microtubule plus-end, promoting thus microtubule polymerization and stabilization [246]. Interestingly, inhibiting the GSK3β (glycogen synthase kinase 3β)-mediated phosphorylation of CLASP2 enhanced the EB1–CLASP2 interaction, which in turn led to an increased Na_v_1.5 delivery at the ID of cardiomyocytes and an increased *I_Na_* [245]. Furthermore, Rhett et al. have shown that in addition to its known localization at the gap junction where it interacts with zonula occludens-1 (ZO-1) [247,248], Cx43 also co-localizes with ZO-1 in the zone surrounding the gap junction, conventionally termed as perinexus and that Cx43 but not ZO-1 interact with Na_v_1.5 at this zone in physiological conditions [249]. In vivo and in vitro assays show that Na_v_1.5 expression and function are reduced as a result of Cx43 expression/function decrease, thus giving more evidence that Cx43 is required for a proper Na_v_1.5 function at the ID [250]. 

Importantly, Na_v_1.5 and Cx43 interaction at the perinexus is thought to be mediated by scaffolding proteins SAP97 and Ankyrin G (AnkG) as their interaction has been reported [241,251]. In the cardiovascular system, ankyrins are critical components of ion channels and transporter signaling complexes, and their dysfunction has been linked with abnormal ion channel and transporter membrane organization and fatal human arrhythmias [252]. Although both ankyrin-B (AnkB, encoded by *ANK2*) and ankyrin-G (*ANK3*) have been found to be expressed in the myocardium, only ankyrin-G has been shown to interact with Na_v_1.5 [253]. Specifically, AnkG is necessary for normal expression of Na_v_1.5 and acts as a coordinating signaling center, functionally coupling Na_v_1.5 gating with upstream kinase and phosphatase enzymes and downstream cytoskeletal proteins [110,254]. AnkG is primarily expressed at the ID membrane and T tubules, where it co-localizes with Na_v_1.5 [142]. In vitro, it has been demonstrated that AnkG binds to Na_v_1.5 and that AnkG downregulation impaired the subcellular localization of Na_v_1.5 and reduced the *I_Na_* current amplitude [255,256]. In vivo, Makara and his collaborators have demonstrated that AnkG plays an indispensable role in directing Na_v_1.5 and its regulatory protein CaMKII to the ID [254,257]. Mutational studies have further confirmed that disrupting the binding of AnkG to Na_v_1.5 impairs AnkG dependent targeting of the Na^+^ channel to the ID leading thus to a reduction in *I_N_*_a_ density and cardiac arrhythmias [253,254,258]. A recent study performed by Yang et al. has demonstrated that AnkG, but not AnkB, are expressed at the IDs and that masking Na_v_1.5 binding sites in AnkG using competitive peptides caused a decrease in sodium channel current (*I_Na_*) and targeting defects of the Na^+^ channels to the ID, but not to LM [213]. However, a more recent study by Cavus and collaborators specified that only canonical AnkG isoforms have this regulatory effect on Na_v_1.5 and that noncanonical (giant) AnkG isoforms mediated electrical dysfunction is independent of Na_v_1.5 [259].

Furthermore, AnkG is thought to mediate the interaction between Cx43 and PKP2, thus connecting desmosomal proteins with the molecular complex that captures the microtubule plus-end at the ID, thus allowing for delivery of Na_v_1.5 [244,256,260]. This is consistent with the fact that loss of desmosomal integrity impacts cardiac conduction and leads to cardiac arrhythmias [260,261,262]. Accordingly, loss of Plakophilin-2 (PKP2), a crucial component of the cardiac desmosome, has been demonstrated to decrease *I_Na_* in cardiac myocytes [263]. Similarly, loss of PKP2 expression in HL1 cells and in induced pluripotent stem cell-derived cardiomyocytes (iPSC-CMs) from a patient with PKP2 deficiency reduced *I_Na_* amplitude [261,264]. Likewise, Rizzo et al. have demonstrated that desmoglein-2 (Dsg2), another desmosome protein, physically interacts with Na_v_1.5 at the ID of mouse cardiomyocytes in vivo [265]. They showed that mice models over-expressing a desmoglein-2 mutation present a wider intercellular space at the level of the ID, longer ventricular activation time, lower conduction velocity, lower upstroke velocity, and lower *I_Na_* amplitude compared to wild type. Although no evidence of direct interaction between Desmoplakin (DSP) and Na_v_1.5 has been reported, RNAi-based Desmoplakin silencing in vitro resulted in a reduction in Na_v_1.5 expression at the ID of cardiomyocytes, an abnormal sub-cellular distribution of Cx43 and Na_v_1.5, *I_Na_* decay, and slowed conduction velocity suggesting that DSP regulates Na_v_1.5 [266].

Similarly, AnkG is established as an adaptor protein that organizes, transports, and anchors Na_v_1.5 to the actin/spectrin cytoskeleton [267,268,269]. In fact, the AnkyrinG-Na_v_1.5 complex is believed to connect with the actin/α-spectrin cytoskeleton through CaMKII-β_IV_-spectrin interaction where the latter acts as a CaMKII-anchoring protein and thereby orchestrating the whole macromolecular complex; however, no evidence of direct interaction between Na_v_1.5 and β_IV_-spectrin has been found yet [255]. On the other hand, β_IV_-spectrin is assumed to control the CaMKII-dependent regulation of Na_v_1.5 at the ID, and loss of β_IV_-spectrin/CaMKII interaction precludes CaMKII-dependent phosphorylation of Na_v_1.5 at Serine 571 in the DI–DII linker and abolishes the stress-induced activation of the pathogenic *I_Na,L_* [270,271]. 

Remme’s team [272] has recently demonstrated that ID Na_v_1.5 physically interacts with coxsackie and adenovirus receptor (CAR), a single-pass transmembrane cell adhesion molecule (CAM) [273]. Furthermore, they have demonstrated that CAR haploinsufficiency decreased *I_Na_* amplitude at the ID, which in turn reduced sodium channel availability at this cell membrane compartment. Na_v_1.5–CAR interaction is only beginning to be understood, and thus, mechanisms underlying this interaction are still to be studied. 

Our current understanding regarding the Na_v_1.5 auto-regulation is still limited. Over the last decades, several controversial studies emerged regarding the sodium channel α-α-subunits interaction and dimerization. However, Clatot and co-workers settled this controversy by demonstrating for the first time that trafficking-defective Na_v_1.5 exerts a dominant-negative effect on non-defective ones through α-α-subunits physical interaction at their N-terminal regions, precluding thus their cell surface expression [274]. Building on these findings, the team further evidenced that cardiac sodium channel α-subunits assemble as dimers with coupled gating and that this dimerization is mediated through an interaction site found within the DI-II linker of Na_v_1.5, between amino acids 493 and 517 [275]. Curiously, earlier studies have shown that 14-3-3 protein, a member of highly conserved cytosolic acidic proteins, physically interacts with the DI-II linker of Na_v_1.5 (between amino acid 417 and 467) at the ID and that this interaction facilitates the dimerization of cardiac sodium channels [276]. Strikingly, Clatot et al. identified a second 14-3-3 protein-Na_v_1.5 interaction site between amino acid 517–555 and demonstrated that co-operative gating behavior but not dimerization of α-subunits is dependent on 14-3-3-Na_v_1.5 interaction [275]. 

##### Na_v_1.5 and the Lateral Membrane’s Interactome

Na_v_1.5 targeting to the LM has been demonstrated to be mediated by the syntrophin–dystrophin complex [3,241]; however, a sub-pool of Na_v_1.5 at the LM, which is independent of syntrophin, has been recently characterized as well [277]. Dystrophin is known to indirectly mediate Na_v_1.5 expression at the LM through binding to Syntrophin adapter protein which physically associates to the PDZ domain-binding motif at the C-terminal region of Na_v_1.5 [3,241,278,279,280]. Interestingly, Matamoros et al. demonstrated that α1-syntrophin also interacts with the N-terminal region of Na_v_1.5 through an “internal” PDZ-like binding domain localized at this region which acts as “chaperone-like” domain that increases Na_v_1.5 density at the LM and *I_Na_* [281]. The same mechanism has been validated for Kir2.1 and Kir2.2 that were demonstrated to reciprocally interact with Na_v_1.5 channels and modulate each other’s trafficking and expression [281,282]. 

Interestingly, Na_v_1.5 has been demonstrated to interact with CASK (calcium/calmodulin-dependent serine kinase), a member of the MAGUK protein family [283]. In several ways, CASK is considered an unconventional Na_v_1.5 regulator since it is the only MAGUK protein that is lateral membrane-specific and also the only Na_v_1.5 interacting protein that exerts a repressive effect on the functional expression of Na_v_1.5, most likely by preventing its early trafficking to the LM. In this regard, CASK has been demonstrated to decrease *I_Na_* when the former is over-expressed and to increase *I_Na_* when CASK is inhibited in vivo and in vitro [283]. 

In addition, Na_v_1.5 has been evidenced to interact with members of the Z-line scaffolding protein complex, such as α-actinin-2 and telethonin. While α-actinin-2 is currently known to physically interact with Na_v_1.5 through the channel DIII–DIV linker [284], the telethonin interaction site on Na_v_1.5 has not yet been identified [101]. α-actinin-2 is thought to positively regulate Na_v_1.5 by increasing its cell surface expression, most likely through promoting its anchoring to the contact zones between T-tubules and Z-lines and connecting the channel to the actin cytoskeleton network [284]. However, scarce information is available regarding the mechanism of Na_v_1.5 regulation by telethonin, although physical interaction between TCAP and Na_v_1.5 was evidenced by co-immunoprecipitation methods and mutations in the telethonin coding gene (*TCAP*) has been found to alter the channel-gating properties of Na_v_1.5 in patients with abnormal gut motility and Brugada syndrome [285,286]. 

Moreover, the role of fibroblast growth factor homologous factors (FHFs), a subset of the fibroblast growth factor (FGF) family [287], has been well elucidated modulating the neuron voltage-gated sodium channels [288]. However, their role in controlling cardiac sodium channel function is still poorly understood and subject to debate. In this respect, fibroblast growth factor homologous factor 1B (FHF1B), also known as FGF12B, has been reported to regulate the biophysical properties and kinetics of Na_v_1.5 through its physical interaction (amino acids 1773–1832) with the Na_v_1.5 C terminal region [289]. Both in vitro data show that FHF1B interacts with Na_v_1.5, and this interaction results in hyperpolarizing shift in steady-state inactivation of this channel [289]. However, the opposite effect has also been reported where a depolarizing shift in the V1/2 of steady-state inactivation has been attributed to the FHF1B-Na_v_1.5 interaction [290]. Furthermore, FGF13 (FHF2), which is the major FHFs in adult mouse hearts, has been identified as a Na_v_1.5 interacting protein [290]. In the cardiomyocyte, FHF2 co-localizes with distinct Na_v_1.5 pools, i.e., the LM and ID suggesting an important role for FHF2 modulating Na_v_1.5 cell surface expression and function [291]. Like FGF12B, FGF13 physically binds to Na_v_1.5 through the channel’s C terminus region. In vivo, FGF13 knockdown altered Na_v_1.5 function resulting in a decreased *I_Na_* current density, reduced Na_v_1.5 channel availability, slowed Na_v_1.5, and reduced *I_Na_* current recovery from inactivation [290]. This effect of FGF13 is isoform-specific [292]. FHFs have also been implicated in voltage-gated sodium channel trafficking control. In this context, FGF14 has been reported as a modulator of Na_v_1.5 current densities in neurons and in the heart by impairing their biophysical properties or by controlling channel trafficking and cell surface expression in vitro [293].

Furthermore, calmodulin (CaM), a ubiquitous Ca2^+^-sensing protein, has been reported to interact with Na_v_1.5 N- and C-terminal regions [294,295,296,297] and the DIII–IV linker [174,295]. This interaction has been demonstrated to enhance slow inactivation and modulate Na_v_1.5 gating [296], while disruption of CaM binding to Na_v_1.5 decreases channel activity and enhances the propensity for persistent Na^+^ current, all resulting from a switch in the Na_V_ inactivation mechanism [297]. Na_v_1.5–CaM interaction has been further studied in a mutational context related to cardiac sodium channelopathies (See Section 4).

Finally, dipeptidyl peptidase-like protein-10 (DPP10), previously reported as a modulator of K_v_4.3-current kinetics [298], has recently emerged as a new regulator of Na_v_1.5 [299]. In vivo, DPP10 has been reported to modulate Na_v_1.5 current kinetics as well by altering voltage dependence of Na^+^ current and upstroke velocity of the action potential [299]. 

##### The Caveolar Na_v_1.5

Cardiac sodium channels have also been localized to cardiomyocyte caveolae, which are specialized subsarcolemmal membrane compartments enriched in lipids and play a crucial role in vesicular trafficking and protein targeting to the cell surface [300,301]. Caveolar Na_v_1.5 is exposed to a very rich macromolecular complex encompassing fatty acids, ion channels (pacemaker channels, potassium channels, calcium channels, etc.), and signaling complexes (G-protein-coupled receptors, protein kinases, etc.). This microenvironment has been reported to regulate Na_v_1.5 function and membrane expression in a multilayers fashion [301]. 

The first layer is related to the biochemical properties of caveolae itself as a specialized lipid raft rich in fatty acids. In this regard, previous reports demonstrated that Na_v_1.5 is blocked by polyunsaturated fatty acids (PUFAs), suggesting that interaction of Na_v_1.5 with the caveolar lipids that also include PUFAs might have the same effect [302,303]. Nonetheless, the mechanism by which caveolar lipid rafts regulate Na_v_1.5 is not yet fully understood. 

The second layer of caveolar Na_v_1.5 regulation is mediated by caveolins which are the major proteins of caveolae [301]. This mechanism was first reported by the Shibata group, which demonstrated that in addition to the indirect β-adrenergic regulation of Na_v_1.5, which is PKA-dependent, stimulation of the β-adrenergic pathway in the presence of a PKA inhibitor, activates G-protein (Gsα) cascade, which in turn leads to a rapid increase of *I_Na_* [300]. A subsequent study by the same group suggested that caveolar Na_v_1.5 channels are stored at caveolae invaginations and that PKA-independent Gsα-dependant stimulation of the β-adrenergic pathway leads to the opening of caveolae, the exposition of Na_v_1.5 channels to the extracellular environment, which in turn increase *I_Na_* [304]. This mechanism has been completely neutralized by anti-caveolin 3 antibodies dialyzed into the myocytes suggesting that caveolar Na_v_1.5 function is dependent on the Gsα-Caveolin 3 (Cav3) interaction [304]. Although Na_v_1.5 has been confirmed to interact with caveolin 3 in rodent and human cardiomyocytes [300,305], it is not yet clear if this interaction is direct or indirect. Several reports suggested that Cav3 modulates Na_v_1.5 function indirectly through inhibiting the nNOS, which is a part of the Na_v_1.5-SNTA1-PMCA4b macromolecular complex [305,306]. As mentioned earlier in this review, a decay in Cav3 expression has been demonstrated to activate S-nitrosylation of Na_v_1.5 through increasing the local NO production, which increased *I_Na,L_* in cardiomyocytes [187]. 

#### 3.1.6. Regulation of Na_v_1.5 Degradation

Maintaining the balance between protein synthesis and degradation is crucial for the fine-tune regulation of Na_v_1.5 levels [307]. In fact, it is currently well established that internalization and degradation of Na_v_1.5 are regulated either by ubiquitination, covalent attachment of ubiquitin moieties [308], or autophagy [309]. The first mechanism is mediated by the interaction of C-terminus PY motifs of Na_v_1.5 with the fourth tryptophan-rich domain (WW) of E3 ubiquitin ligase NEDD4-2, which leads to the labeling of Na_v_1.5 by ubiquitin residues that will be later recognized by the degradation machine [100,310,311]. Interestingly, yeast two-hybrid data demonstrated that the interaction between Na_v_1.5/αβ-Cristallin from one hand and αβ-Cristallin/Nedd4-2 from another hand reduced internalization of cell surface Na_v_1.5 and ubiquitination of Na_v_1.5 [312]. Similarly, serum and glucocorticoid inducible kinase (SGK) has been reported to regulate Na_v_1.5 degradation by phosphorylating and inhibiting Nedd4-2 [313], whereas UBC9, a SUMO-conjugating enzyme, has been shown to promote Na_v_1.5 ubiquitination [314]. A very recent study by Liu et al. demonstrated that Na_v_1.5 ubiquitination would be downregulated by the association of FAT10, a small ubiquitin-like modifier, to the C-terminal lysine residues of Na_v_1.5, thus decreasing the binding of Na_v_1.5 to the Nedd4-2 and preventing its degradation [315].

Nedd4-2 has been reported as a direct target of AMP-activated protein kinase (AMPK) in epithelial cells [316]. However, a recent report by Liu X et al. attributed a Nedd4-2 independent Na_v_1.5 degradation mechanism to AMPK [309]. AMPK, through phosphorylating Na_v_1.5 T101 residue, facilitates the association of the channel to the autophagic adapter protein and microtubule-associated protein 1 light chain 3 (LC3) and exposes the complex to the autophagic degradation machinery [309]. 

#### 3.1.7. Effect of Gonadal Hormones on Na_v_1.5 Expression and Function

The male predominance of some sodium channelopathies such as Brugada syndrome has been extensively studied over the last few years, thus questioning a possible link between sex hormones and Na_v_1.5 [317,318]. However, comparing the expression levels of Na_v_1.5 between normal male and female human hearts showed no difference [319]. In addition, concentration-related block of Na_v_1.5 by estradiol showed that estradiol could not reduce the current of Na_v_1.5 [320], although a slight reduction in *I_Na_* currents has been observed at a high concentration of estradiol in vitro [321]. Yang et al. have recently studied the expression levels and function of Na_v_1.5 in HEK293 cells co-expressing *SCN5A* (wild-type or BrS mutants R878C and R104W) and sex hormone receptors. They whereby showed that sex hormones have no effects on the expression level of *SCN5A* (either WT or mutant) and *I_Na_* currents [322]. Similarly, gonadal hormones testosterone and estrogen showed no effect on fast *I_Na_* in a canine model [323]. However, a recent study by Hu et al. demonstrated that estrogen through its rapid signal receptor GPER ameliorated the damaging effects of stress in human induced pluripotent stem cell-derived cardiomyocytes (hiPSC-CMs) model mimicking β-adrenergic overstimulation [324]. Taken together, these findings demonstrate that our current knowledge on the regulation of Na_v_1.5 by sex hormones is still limited and that further studies in this regard are necessary. 

#### 3.1.8. Effect of Temperature and pH on Na_v_1.5 Expression and Function

Febrile states and acidosis are two environmental factors that have been extensively studied as non-genomic modulators of Na_v_1.5 function in health and disease. It is currently well known that Na_v_1.5 kinetic is temperature and pH-sensitive [325]. In this context, mild hypothermia has been described as an antiarrhythmic factor that maintains myocardial conduction during prolonged ischemia by sustaining Na_v_1.5 and Cx43 function [326], whereas hyperthermia has been described as a proarrhythmic factor, especially in combination with *SCN5A* mutations as is the case in Brugada syndrome [327,328,329,330]. Two mechanisms have been suggested so far for the temperature-dependent regulation of Na_v_1.5. The first one is a direct mechanism by which temperature accelerates the inactivation of only the wild-type Na_v_1.5 channels in heterozygous patients, which results in the misbalance between depolarization and repolarization currents and thus may lead to fever-induced arrhythmias [40,331]. The second mechanism is indirect by which temperature modulates the function of Na_v_1.5 interacting proteins, which in turn modulate Na_v_1.5 function as is the case of FGF13 [332].

Similarly, fluctuation of the extracellular pH has been demonstrated to influence the Na_v_1.5 function. For instance, acidic extracellular pH has been shown to modify wild-type Na_v_1.5 kinetics by destabilizing both the fast inactivated and the slow inactivated states of Na_v_1.5 [333]. In addition, it has been reported that extracellular protons disrupt charge immobilization which leads to the destabilization of the Na_v_1.5 fast-inactivation through direct interaction with outer ring carboxylates of the Na_v_1.5 DIII or DIV [334]. Particularly, His-880 and Cys-373 were identified as the key mediator of Na_v_1.5 sensitivity to pH fluctuation, where Cys-373 is responsible for isoform-specific proton modulation of use-dependent inactivation of Na_v_1.5 [335]. 

## 4. Cardiac Sodium Channelopathies

*SCN5A* dysfunction has been extensively reported in distinct cardiac channelopathies such as long QT syndrome, Brugada syndrome, atrial fibrillation, ventricular fibrillation, sick sinus syndrome, and sudden infant death syndrome, as well as in complex electrophysiological disorders that combine several of the previously mentioned channelopathies. In the next paragraphs, we provide an updated view of the mutational, genomic, and non-genomic contribution to *SCN5A* dysfunction on each of these channelopathies (Table 1).

### 4.1. Long QT Syndrome

Seminal studies by Wang et al. [336] determined a causative link between *SCN5A* mutations and long QT syndrome (LQT3). These authors also determined the biophysical and functional characteristics of the novel identified mutations that displayed non-inactivating *I_Na_* amounting to a few percent of the peak inward *I_Na_*, as well as impairment on voltage dependence and rate of inactivation and the rate of recovery from inactivation [337]. Since these early studies, more than 80 different mutations have been identified in *SCN5A* associated with LQT, accounting for 5–10% of the cases (see for recent reviews [14,338,339,340,341]). Functional analyses of several of the identified LQT *SCN5A* variants displayed gain-of-function either by increasing the late phase of the *I_Na_* or increasing the window current or both conditions simultaneously, yet our current understanding of the functional roles of most *SCN5A* mutations described remains elusive. 

In addition to mutations on *SCN5A* associated with long QT syndrome, several *SCN5A*/Na_v_1.5 interacting proteins have also been associated in this context. Mutations in the sodium channel ancillary protein *SCN1B* are associated with LQT, leading to increased *I_NaL_* [342], while mutations in *SCN4B* have also been identified [231], yet its plausible implication in LQT is still disputed [343]. Besides these ancillary subunits, a large number of proteins have been reported to interact with *SCN5A*/Na_v_1.5, as recently reviewed by Abriel Hugues [101] and detailed in the previous subchapters of this manuscript. In this context, mutations in syntrophin (*SNTA1*) and caveolin (*Cav3*) are associated with long QT and disrupt sodium channel function, increasing the *I_NaL_* [186,187,344,345,346,347]. On the other hand, mutations in *BAG3*, ankyrin B (*ANKB*), and α-actinin (*ACTN1*) leads to LQT, but it remains unclear how such mutations affect the *I_Na_* [294,348,349], while calmodulin (*CaM*) and telethonin (*TCAP*) have been implicated in *SCN5A*/Na_v_1.5 interaction and function [163,285] yet to date no mutations have been reported in the context of LQT syndrome.

The genomic contribution to long QT syndrome in transcriptional regulators of *SCN5A* expression is still incipient since only variants on *TBX5* have been reported but not in any of the other transcriptional modulators. Variants affecting transcriptional regulators influencing *SCN5A* expression have been recently linked to long QT syndrome. In particular, Markunas et al. [350] described a *TBX5* variant that co-segregated with prolonged QT interval in a family with otherwise genotype-negative LQTS and demonstrated that such variant impaired the transactivation capacity of this transcription factor. Nieto-Marín et al. [351] reported two additional *TBX5* variants that co-segregated with LQT and BrS patients and electrophysiologically impaired *I_Na_* currents in vitro. 

Several *SCN5A* mutations leading to splice donor variants have been associated with LQT [339,352,353,354,355,356,357]; however, to date, the functional relationship between the distinct *SCN5A* isoforms and this syndrome remains to be elucidated. While our current understanding of distinct non-coding RNAs in cardiovascular pathology is increasingly emerging, no data are available regarding the functional contribution of microRNAs and/or lncRNAs to long QT syndrome physiopathology. Similarly, the contribution of post-transcriptional *SCN5A*/Na_v_1.5 regulation by phosphorylation, glycosylation, acetylation, and/or methylation to QT syndrome has not been reported, and the contribution of ubiquitination is currently controversial [358,359]. Importantly, acetylation in *KCNH2*, i.e., another ion channel that, if mutated, contributes to long QT syndrome, has been reported [360], opening the possibility that *SCN5A*/Na_v_1.5 post-transcriptional regulation also contributes to LQT.

### 4.2. Brugada Syndrome

*SCN5A* mutations were associated for the first time to the right bundle branch and ST-elevation syndrome, i.e., Brugada syndrome by Chen et al. [25]. These authors identified a two-nucleotide insertion after the first four nucleotides of the splice donor sequence in intron 7, leading to impaired *SCN5A* splicing. They also identified a deletion of a single nucleotide in the *SCN5A* gene that resulted in the elimination of two transmembrane domains and the C-terminal portion of the cardiac sodium channel. Since then, an increasing number of *SCN5A* mutations have been reported to be associated with Brugada syndrome. To date, more than a hundred mutations have been reported in BrS. Functional analyses of several of these *SCN5A* reported mutations to demonstrate in most cases, a loss-of-function is achieved, either by decreased Na_v_1.5 expression in the sarcolemma, because the channels are non-functional or because there is an impaired gating off the channel that results in decreased *I_Na_* current. However, only a relatively limited number of *SCN5A* mutations associated with BrS have been fully electrophysiologically characterized.

Importantly, single point mutations have also been associated with both long QT and BrS phenotypes [26,361], and several of these have been electrophysiologically characterized [24,362,363,364,365], yet the precise molecular mechanism of their dual action remains enigmatic.

As previously reported in the context of LQT, in addition to mutations on *SCN5A*, several *SCN5A*/Na_v_1.5 interacting proteins have also been associated with Brugada syndrome. Mutations in the sodium channel ancillary proteins SCN1B [232,356,359,366,367,368,369,370], *SCN2B* [229], and *SCN3B* [356] are associated with Brugada syndrome, leading in several cases to decrease *I_Na_* current [366] and/or cellular trafficking defects [229,356]. Besides the role of the sodium channel ancillary subunits, additional *SCN5A*/Na_v_1.5 interacting proteins have been reported in BrS. Mutations in Plakophilin *(PKP2)* [260,371] *MOG1* [9,201,372,373,374,375], *FGF13* [376], syntrophin *(SNTA1)* [377], *NEDD4* [378,379], Tmem168 [379] and telethonin [285,286] are identified in BrS patients and their implication to sodium channel function has been reported. Distinct Na_v_1.5 interacting protein mutants lead to *I_Na_* deficit [261,286,375,377] while others influence Na_v_ trafficking and thus subcellular localization [201,379].

On the other hand, mutations in desmoglein and *SAP97* have been described in BrS patients [380,381], but their influence on *SCN5A*/Na_v_1.5 remains to be elucidated, while calmodulin, CamKII, and *GPD1L* modulation of *SCN5A*/Na_v_1.5 is well-established [157,163,382,383], but to date, no mutation in these genes have been reported in the context of Brugada syndrome. 

At the transcriptional level, Nieto-Marín [351] identified *TBX5* variants associated with BrS and LQT, as previously mentioned. Furthermore, additional evidence of the transcriptional modulation of *SCN5A* expression in Brugada syndrome, including *GATA4* [384] and *IRX3* [385] variants, have been reported. Transcriptional contributions by mutations in the *SCN5A* promoter are also linked to Brugada syndrome [384], causing decreased *SCN5A*/Na_v_1.5 and *I_Na_*, thus loss-of-function. Importantly, they have also been associated with distinct arrhythmogenic diseases [24,386]. More recently, [387] reported that a common *SCN5A* polymorphism, i.e., H558R, modulates the *SCN5A* promoter methylation and thus the clinical phenotype of Brugada syndrome patients. However, it remains unclear which are the molecular mechanisms underlying such association.

The importance of non-coding RNAs in the context of Brugada syndrome has been recently explored using an integrative omics approach [388]. These authors identified several microRNAs that are distinctly upregulated in BrS, such as miR-92a-3p and miR-320b, or down-regulated such as miR-425-5p and established their plausible links as molecular determinants of Brugada syndrome, yet functional evidence is missing. More recently, Matsumura et al. [93] described *SCN5A* coding variants that lack genotype-phenotype concordance, and additionally, *SCN5A* 3’UTR variants were identified that impaired microRNA binding sites but similarly failed to properly segregate with BrS phenotype [93], suggesting that a combination of multiple genetic factors, rather than a single variant is the cause of BrS onset [93]. Thus, to date, the plausible role of ncRNAs in BrS remains almost unexplored.

Our current understanding of the mechanistic links between *SCN5A*/Na_v_1.5 post-transcriptional modifications and the onset of Brugada syndrome is scarce. Aiba et al. [389] described that R526H and S528A *SCN5A* mutations, identified in a BrS family, impaired Na_v_1.5 phosphorylation, leading to reduced peak current densities, yet steady-state activation, inactivation, and recovery for inactivation were not modified. Besides this study, no additional links for glycosylation, acetylation, and/or methylation have been reported in the context of BrS.

### 4.3. Atrial Fibrillation

Atrial fibrillation has also been associated with mutations in *SCN5A*. Ellinor et al. reported missense mutation in a 45-year-old male proband and his affected father among a series of 57 probands with a familial history of isolated or ‘lone’ atrial fibrillation [384]. More recently, Darbar et al. [390] identified eight heterozygous variants in ten probands that were not found in age-, sex-, and ethnicity-matched controls. In addition, rare nonsynonymous coding region variants previously reported were also demonstrating that in their study, nearly 6% of AF probands carried heterozygous mutations or rare *SCN5A* variants. Thus, the causal contribution of *SCN5A* to familial AF is limited. 

Curiously, an extensive array of *SCN5A* mutations have been associated with atrial fibrillation and other electrophysiological disorders such as BrS [391], cardiac conduction defects [392], LQT [393], and even a spectrum of atrial flutter, conduction diseases, and BrS [394]. 

Mutations in all sodium channel ancillary subunits have been reported in atrial fibrillation patients [395,396,397,398], while only mutations in Mog1 [375,399], ankyrin [400,401], alpha-actinin [402], and caveolin [403] have been associated with atrial fibrillation among those other *SCN5A*/Na_v_1.5 interacting proteins. Curiously, no evidence of their functional impact on *SCN5A*/Na_v_1.5 function is reported to date. 

### 4.4. Ventricular Fibrillation

Mutations in *SCN5A* have been associated with ventricular fibrillation [404,405,406] and idiopathic ventricular fibrillation [407,408,409]. However, a large array of *SCN5A* mutations linked to ventricular fibrillation are reported in the context of additional cardiac impairments, such as Brugada syndrome [410,411,412,413], atrial fibrillation [414], acute myocardial infarction [415,416,417], sudden cardiac death [418,419], or Graves’ disease [420]. Surprisingly, while abundant information is available about the genetic determinants of ventricular fibrillation, scarce information is available about the molecular mechanisms leading to ventricular fibrillation. 

Genetic screening of sodium channel ancillary subunits has revealed only mutation in *SCN3B* associated with ventricular fibrillation [421]. Although no direct evidence of mutations in desmosomal proteins such as desmoglein or plakoglobin has been reported in ventricular fibrillation, there is compiling evidence that such mutations in the context of arrhythmogenic right ventricular cardiomyopathy (ARVD) predispose to an early onset of ventricular fibrillation [422,423,424,425]. In addition to those *SCN5A*/Na_v_1.5 interacting proteins, mutations in calmodulin [426,427] and alpha-actinin [428] have also been reported in ventricular fibrillation patients, yet their plausible implications deregulating *SCN5A*/Na_v_1.5 is not reported to date. 

Within the currently described transcriptional regulators of *SCN5A* expression, only mutations in *IRX3* have been associated with ventricular fibrillation [65]. Interestingly, those *IRX3* mutations impaired *SCN5A* expression in in vitro experimental assays, yet the causal contribution to ventricular fibrillation remains unclear. On the other hand, no evidence has been reported so far on *SCN5A*/Na_v_1.5 post-transcriptional modifications in the context of ventricular fibrillation.

### 4.5. Sick Sinus Syndrome

Ample evidence has been reported on *SCN5A* mutations leading to sick sinus syndrome [29,429,430,431,432,433,434,435,436] alone or in combination with other electrophysiological disorders such as atrial fibrillation [437] and Brugada syndrome with conduction diseases [30]. However, no mutations in sodium channels ancillary subunits or *SCN5A*/Na_v_1.5 interacting proteins have been reported. Similarly, no link between *SCN5A* transcriptional and post-transcriptional regulators and sick sinus syndrome is reported to date.

### 4.6. Sudden Infant Death Syndrome

Mutations in *SCN5A* are also causative of sudden infant death syndrome [438,439,440,441,442,443,444] as well as in channel ancillary subunits [445,446,447,448]. It is assumed that, as in the case of Brugada syndrome, an *SCN5A*/Na_v_1.5 loss-of-function underlies sudden infant death. In this context, Tan et al. [446] described *SCN3B* and *SCNB4* mutations that decreased peak *I_Na_* current but increased *I_NaL_*, whereas Neubauer et al. [412] and Denti et al. [447] identified a novel *SCN1B* mutations that decreased *I_Na_* density, providing thus plausible electrophysiological mechanisms underlying sudden infant death syndrome. Importantly *SCN5A* mutations in SIDS have also been identified in conjunction with other electrophysiological disorders, such as Brugada syndrome [356].

In addition to those mutations, several *SCN5A*/Na_v_1.5 interacting proteins have also been associated with sudden infant cardiac syndromes, such as calmodulin [383,449], syntrophin [186,346,450], and caveolin [187,451]. Cheng et al. reported that syntrophin mutation leads to increased peak *I_Na_* current and overlap between activation and inactivation curves increasing thus the window current [186]. Similar findings on the increased peak *I_Na_* current were also reported by Wu et al. [346] and Cheng et al. [450], a phenotype that was rescued by another calmodulin variant identified in SIDS. Caveolin mutations seem to distinctly contribute to sodium regulation, either by suppressing the *I_NaL_* by inhibiting nNOS-dependent S-nitrosylation of *SCN5A* [187] or by the persistence of the *I_NaL_* [451].

On the other hand, no association between *SCN5A* transcriptional and post-transcriptional modulators and sudden infant death syndrome has been reported to date. 

### 4.7. Other Channelopathies

*SCN5A* mutations also play a pivotal role in complex electrophysiological disorders that present a combination of alterations, particularly on conduction disorders associated with sick sinus syndrome, atrial fibrillation and ventricular tachycardia [435]; LQT and BrS [452]; atrial fibrillation, BrS, and sudden cardiac death [394]; BrS [453]; BrS and AF [454]; or atrial arrhythmias [392]. Similarly, increasing evidence is also reported in cases of sudden cardiac death, alone [455] or in combination with LQT and dilated cardiomyopathy [452], ventricular fibrillation and BrS [456], or atrial fibrillation [433]. Our current understanding of the molecular mechanisms providing such diversity of phenotypic manifestations is still limited. It is important nonetheless to highlight that mutations in *SCN5A*/Na_v_1.5 interacting proteins [9,201,401] and/or transcriptional regulators [457] have also been recently reported to be associated with these complex electrophysiological disorders.

**Table 1:** Summary of the Na_v_1.5-interacting protein-coding genes involved in cardiac sodium channelopathies.

Altered Gene/Alteration	Effect on *I_Na_* Current	Mode of Action	References
**Long QT syndrome**			
*SCN5A*	Increased *I_NaL_*, increased window current	NA	[14,336,338,339,340,341]
*SCN1B*	Increased *I_NaL_*	Ancillary subunit	[342]
*SCN4B*	Undetermined	Ancillary subunit	[231,343]
*SNTA1*	Increased *I_NaL_*	Activation of nNOS–*SCN5A* macromolecular complex	[186,187,344,345,346,347]
*Cav3*	Increased *I_NaL_*	nNOS-dependent S-nitrosylation of *SCN5A*	[187]
*BAG3*	Undetermined	Undetermined	[348]
*ANK B*	Undetermined	Undetermined	[258]
ACTN1	Undetermined	Undetermined	[349]
*TBX5*	Increased *I_NaL_*	Transactivation impairment	[350,351]
**Brugada syndrome**			
*SCN5A*	Decreased *I_Na_*	NA	[24,25,26,361,362,363,364,365]
*SCN1B*	Decreased *I_Na_*	Ancillary subunit; cellular trafficking defects	[232,356,359,366,367,368,369,370]
*SCN2B*	Decreased *I_Na_*	Ancillary subunit; cellular trafficking defects	[229]
*SCN3B*	Decreased *I_Na_*	Ancillary subunit; cellular trafficking defects	[356]
*PKP2*	Decreased *I_Na_*	*I_Na_* deficit	[260,371]
*MOG1*	Decreased *I_Na_*	*I_Na_* deficit; subcellular trafficking	[9,201,372,373,374,375]
*FGF13*	Decreased *I_Na_*	Enhanced Na_v_1.5 inactivation	[376]
*SNTA1*	Decreased *I_Na_*	Defective Na_v_1.5 protein interaction	[377]
*TMEM168*	Decreased *I_Na_*	Reduced Na_v_1-5 expression	[378,379]
*TCAP*	Decreased *I_Na_*	Defective Na_v_1.5 protein interaction	[285,286]
*SAP97*	Undetermined	Undetermined	[380,381]
*TBX5*	Undetermined	Transactivation impairment	[351]
*GATA4*	Undetermined	Undetermined	[384]
*IRX3*	Undetermined	Undetermined	[385]
*SCN5A promoter methylation*	Undetermined	Decreased *SCN5A* promoter methylation	[387]
*SCN5A promoter mutations*	Undetermined	Transactivation impairment	[384]
*Na_v_1.5 phosphorylation*	Reduced peak *I_Na_* density	Impaired PKA stimulation	[389]
**Atrial fibrillation**			
*SCN5A*	Undetermined	NA	[384,390]
*SCN1B*	Reduced *I_Na_*	Ancillary subunit; altered channel gating	[395]
*SCN2B*	Reduced *I_Na_*	Ancillary subunit; altered channel gating	[395]
*SCN3B*	Undetermined	Ancillary subunit	[398]
*SCN4B*	Undetermined	Ancillary subunit	[396,397]
*MOG1*	Reduced *I_Na_*	Undetermined	[375,399]
*ANK*	Undetermined	Undetermined	[400,401]
*ACTN1*	Undetermined	Undetermined	[402]
*Cav3*	Undetermined	Undetermined	[403]
**Ventricular fibrillation**			
*SCN5A*	Reduced *I_Na_*	*NA*	[404,405,406]
*SCN3B*	Reduced peak *I_Na_*	Ancillary subunit; impaired trafficking	[421]
*CaM*	Undetermined	Undetermined	[426,427]
*ACTN1*	Undetermined	Undetermined	[428]
*IRX3*	Undetermined	Impaired transactivation	[65]
**Sick sinus syndrome**			
*SCN5A*	Reduced *I_Na_*	NA	[29,429,430,431,432,433,434,435,436]
**Sudden infant death syndrome**			
*SCN5A*	Increased *I_NaL_*	Impaired Na_v_1.5 inactivation	[438,439,440,441,442,443,444]
*SCN1B*	Decreased *I_Na_* density	Ancillary subunit	[447]
*SCN3B*	Decreased *I_Na_*, Increased *I_NaL_*	Ancillary subunit	[446,448]
*SCN4B*	Decreased *I_Na_*, Increased *I_NaL_*	Ancillary subunit	[446]
*CaM*	Undetermined	Undetermined	[383,449]
*SNTA1*	Increased peak *I_Na_* and window current	Undetermined	[186,346,450]
*Cav3*	Suppression of *I_NaL_*	Inhibiting nNOS-dependent S-nitrosylation of Na_v_1.5	[187,451]

## 5. Conclusions and Perspectives

Understanding the complex cellular and molecular mechanisms by which the cardiac sodium channel is formed is essential for dissecting and eventually repairing the culprits of an important array of cardiac arrhythmogenic defects with a large impact on society, such as sudden death. In this review, we have provided current state-of-the-art information of the molecular events that emanate with the transcription and transcriptional regulation of the *SCN5A* gene, the post-transcriptional modifications that the *SCN5A* transcript undergoes leading to the configuration of a large array of distinct alternative spliced variants and being subjected to several types of epigenetic modulations along the way. Subsequently, distinct interacting proteins accompany the nascent Na_v_1.5 protein along with different subcellular organelles until it reaches its final membrane destination, accumulating a large number of diverse post-translational modifications, which eventually allows to carry out a fundamental electrophysiological role on the configuration of the cardiac action potential. Importantly, defects on a large number of these pathways have a tremendous impact on Na_v_1.5 functionality and are thus intimately linked to cardiac arrhythmias. While the advancement of the knowledge of the cardiac sodium channel biology and electrophysiology has greatly increased over the last two decades, understanding of the functional contribution of multiple *SCN5A* mutations as well as mutations in *SCN5A*/Na_v_1.5 interacting proteins is still incipient. In this context, it is particularly challenging to deeply understand the molecular mechanisms driving the association of single mutations with distinct impaired electrophysiological entities, such as Brugada and LQT syndrome, or in cases of even more complex phenotypical manifestations, such as sick sinus syndrome, atrial fibrillation, and ventricular tachycardia. In the coming years, insights into distinct molecular mechanisms involved in *SCN5A*/Na_v_1.5 formation and maturation will be more deeply explored, providing us with an increased understanding of the molecular and cellular routes that, if impaired, lead to distinct cardiac electrophysiological pathophysiologies, particularly on our currently poorly explored such as ventricular fibrillation, sick sinus syndrome, and sudden infant cardiac death. We also envision that the impact of non-coding biology into the modulation of *SCN5A*/Na_v_1.5 will progressively emerge, providing novel therapeutic tools to repair or at least modulate the pathological consequences of severe arrhythmogenic defects such as long QT, Brugada syndrome, and/or atrial fibrillation.

## Abbreviations

AP	Action potential
VS	Voltage-sensing module
PM	Pore module
PUFA	Polyunsaturated fatty acids
ER	Endoplasmic reticulum
RE	Regulatory elements
UPR	Unfolded protein response
ERAD	ER-associated degradation pathway
PTMs	Post-translational modifications
ID	Intercalated discs
LM	Lateral membrane
*I_ks_*	Slow component of the delayed rectifier potassium current
*I_to_*	Transient outward potassium current
